# Freely suspended perforated polymer nanomembranes for protein separations

**DOI:** 10.1038/s41598-018-22200-4

**Published:** 2018-03-13

**Authors:** Christian Schuster, Agnes Rodler, Rupert Tscheliessnig, Alois Jungbauer

**Affiliations:** 1 Austrian Centre of Industrial Biotechnology, Vienna, Austria; 2 University of Natural Resources and Life Sciences, Vienna, Austria

## Abstract

Selective removal of nanometer-sized compounds such as proteins from fluids is an often challenging task in many scientific and industrial areas. Addressing such tasks with highly efficient and selective membranes is desirable since commonly used chromatographic approaches are expensive and difficult to scale up. Nanomembranes, molecularly thin separation layers, have been predicted and shown to possess outstanding properties but in spite ultra-fast diffusion times and high-resolution separation, to date they generally lack either of two crucial characteristics: compatibility with biological fluids and low-cost production. Here we report the fast and easy fabrication of highly crosslinked polymer membranes based on a thermoset resin (poly[(*o*-cresyl glycidyl ether)-*co*-formaldehyde (PCGF) cured with branched polyethyleneimine (PEI)) with nanoscale perforations of 25 nm diameter. During spin casting, microphase separation of a polylactide-*co*-glycolide induces the formation of nanometer sized domains that serve as templates for perforations which penetrate the 80 nm thick membranes. Ultrathin perforated nanomembranes can be freely suspended on the cm scale, exhibit high mechanical strength, low surface energies and a sharp permeability cutoff at a hydrodynamic diameter of 10 nm suitable for protein separations.

## Introduction

Permeable membranes with nanoscale thickness, defined pore geometries and sizes, namely perforated nanomembranes, are the ideal separation materials of tomorrow’s industry as they combine high mass transfer with sharp cut-off consequently enabling fast and high-resolution separations. Currently, membranes applied in biotechnology are predominantly used for concentration by ultrafiltration or buffer exchange by ultra-diafiltration^1,2^. They are rarely used for protein separations where chromatographic processes occupy a seemingly indispensable position. Hindrance theory as well as experimental evidence suggest that the performance of separation membranes improves with decreasing thickness. This is especially the case when the thickness of the separation layer approaches its nominal pore size and the size of the molecules being separated^3^. Hence, porous nanomembranes represent an alternative or complementary material for protein purification by high resolution or group separations with the potential to induce a paradigm shift in separations technology. Since peptide purification for pharmaceutical formulation has become the major cost driver such highly efficient nanomembrane based separations bear enormous economic potential. However, this has not yet been widely adopted by the industry^1,4^. In pharmaceutical biotechnology and biomanufacturing the use of disposables and single use separation materials is a current trend^5,6^ where a high degree of quality but still reasonable costs are a prerequisite. For nanomaterials the costs associated with the fabrication process clearly dominate material costs and scalable, economically feasible fabrication is sought with considerable effort^4^.

Perforated nanomembranes have been produced by numerous groups and many are derived from inorganic silicon based materials^4,7–9^ with very regular structures. Various different approaches have been developed in the past decade to impart such nanoscale patterns to different materials. For instance, self-assembly of nanoparticles as templates^10,11^ and self-organizing polymers^12–14^ have been used to generate nanoscale patterns acting as processing masks to eventually generate perforations in thin sheets of material. While the geometries are excellently suited for separation of proteins and other biomolecules, these inorganic materials have the drawback of poor compatibility with biological fluids and the contained proteins. A high surface free energy of such materials leads to proteins intercalating and thus adsorbing at the water-solid interface to reduce the overall energy of the system^7,15,16^. Similarly, hydrophobicity of some materials poses a threat to actual applicability due to biomolecule adsorption and fouling^16^. Self-organization of block copolymers has not only been used to generate masks but also presents a powerful route to generate polymeric membranes with porous structures^17–19^. The length and chemical difference of the polymer subunits can be utilized to govern the resulting structure of the material and its features^20^. Highly size selective and permeable membranes supported by a robust porous substrate were demonstrated for efficient virus filtration with such a method^21^. In another approach, nanoparticle assisted templating has been employed to prepare perforated, pH-stimuli responsive hydrogel-brush nanomembranes which close their pores at low pH values^22^. Despite their high potential, these materials are typically restricted to small lab scale and entail tedious polymer synthesis or lengthy processing steps, hurdles which have to be overcome for industrial production.

Other, polymer-based nanomembranes have been introduced where an epoxy resin (poly[(*o*-cresyl glycidyl ether)-*co*-formaldehyde], PCGF) is cured with branched polyethyleneimine (PEI) to form a continuous non-porous (from here on referred to as dense) and highly crosslinked nanothick (<100 nm thickness) polymer film^23^. The authors showed that, by employing a simple spin coating process, such covalently crosslinked polymeric nanomembranes can be fabricated with aspect ratios of more than 10^6^ for which they coined the term ‘large’^23^ or ‘giant’^24^ nanomembranes. Apart from their intended use for sensor and insulator materials these polymer films may be well compatible with biological fluids and proteins since similar polymers are used for fabrication of chromatography beads and monoliths for protein separation. In fact, PEI is known for improving shelf stability of proteins and protective effects in unfavorable environments^25^. Despite their outstanding combination of mechanical and chemical properties encompassing high tensile strength, chemical resistance, adhesive properties and dimensional stability^23^, thin films derived from thermoset resins have yet to be exploited for the preparation of porous or perforated separation nanomembranes^26^. Such nanomembranes would possess sufficient mechanical and chemical robustness while providing superior biocompatibility and the benefits associated with polymer processing such as fast and low cost processes and scalability.

Phase separation occurring in ternary mixtures due to rapid compositional quenching during spin coating has been studied and used to obtain functional nanostructured thin films in a bottom up fashion^27,28^. An intriguing aspect is that moderate variations of mixture composition or Flory-Huggins interaction parameters make a wide variety of resulting morphologies accessible. They range from meander- and network like structures to well dispersed circular domains with sizes of several µm down to tens of nm. In consequence, it was shown that phase separation is a possibility to introduce voids into thin polymeric films if one component can be removed selectively. Recently, porous freestanding nanomembranes have been cast from a binary mixture of poly(lactic acid) and poly(vinyl alcohol) with a thickness down to ~60 nm and average pore diameters between 65 and 800 nm^29^. The authors showed that the viability of epithelial cells during adherent growth on these perforated nanomembranes was enhanced compared to non-perforated membranes. This effect was attributed to the dimensional similarity of the perforations and the filopodia filaments of the cells. In a follow up study the fabrication procedure has been tuned to yield perforated nanomembranes with an average pore size of 51 nm^30^. These perforations were then used as immobilization sites for ~27 nm large trimers of bacterial outer membrane protein. Thereby the authors prepared ultrathin freestanding separation membranes with passive diffusion selectivity for certain metal ions. Although these membranes, with pore sizes down to ~51 nm, represent an interesting separation material and could potentially also be used for viral clearance purposes or cell removal the pores seem to be too large for the separation of regular proteins with satisfying resolution. Nevertheless, such a polymer based approach is well suited for economic production and scaling to large separation areas given a sufficiently robust material.

In this work, we demonstrate the bottom-up manufacture of nanothick freestanding polymer separation membranes with low cost equipment which is devoid of any complex processing steps. We use a covalently crosslinking thermoset resin in combination with lateral structure formation by phase separation induced during spin coating of a ternary polymer blend. The resulting thin film can be transformed into a self-supporting, perforated polymeric nanomembrane preserving the specific mechanical and chemical properties observed for equivalent dense nanomembranes. The system is well suited to fabricate nanomembranes with perforations of defined geometry for the separation of proteins and the use with biological fluids. Their hydrophilic nature and low surface free energy promotes biocompatibility such that these nanomembranes will be an excellent choice for the development and implementation of single use membrane devices for biotechnological applications.

## Membrane Fabrication and Material Related Characteristics

**Figure 1:**
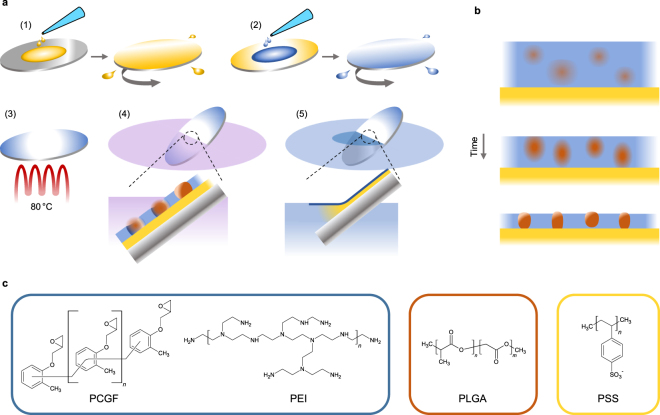
Fabrication of perforated nanomembranes. (**a**) Illustration of the fabrication procedure: (1) Sacrificial layer casting onto polished silicon wafer. (2) Coating of the nanomembrane casting solution containing PEI, PCGF and PLGA dissolved in chloroform onto sacrificial layer. (3) Baking step to crosslink thermoset resin component. (4) Selective dissolution of PLGA by immersion into chloroform to create perforations in the continuous nanomembrane matrix. (5) Immersion into water releasing the nanomembrane from the wafer onto the water surface. (**b**) Schematic of phase separation events occurring during spin coating illustrated at several points in time. (**c**) Chemical structures of the polymers used for nanomembrane fabrication: Thermoset resin constituents poly[(*o*-cresyl glycidyl ether)-*co*-formaldehyde] (PCGF) and branched polyethyleneimine (PEI) comprise the nanomembrane matrix, poly(D,L-lactide-*co*-glycolide) (PLGA) acts as phase separating porogen and polystyrene sulfonate (PSS) is used as a water soluble sacrificial layer.

**Figure 2:**
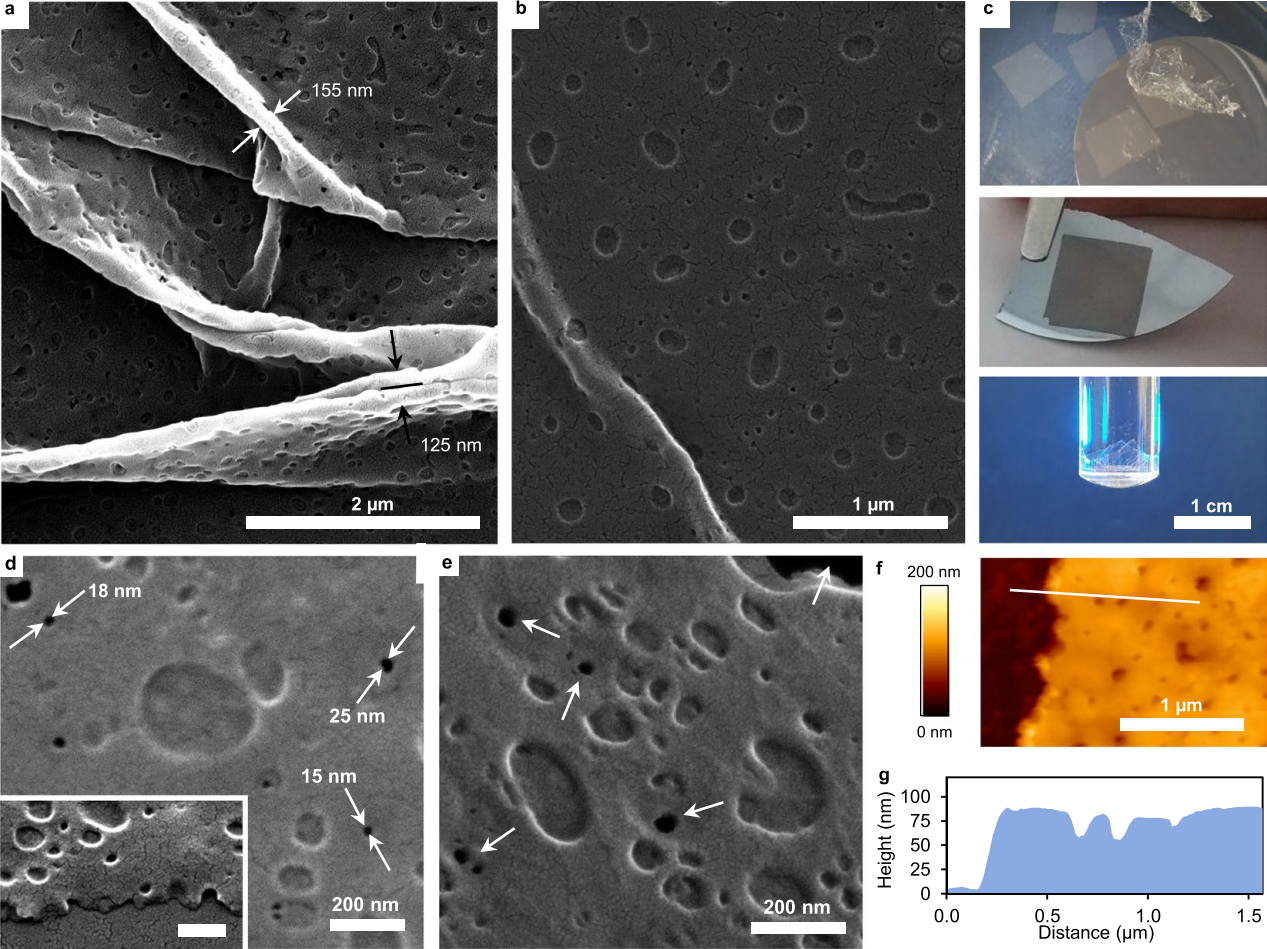
Structural characteristics of perforated nanomembranes. (**a**) SEM image of a warped perforated nanomembrane illustrating the surface topology of pores and depressions. Arrows indicate positions for membrane thickness approximation where it is folded without a gap in between. (**b**) SEM image of a membrane stretched out on a silicon wafer with a small ridge. The difference in length scale of the phase separated domains is clearly visible. (**c**) Photographs of membrane pieces floating on the surface of water (rectangular shapes) and a submerged piece (crumpled shape) (upper image), a membrane picked up on top of a silicon wafer held with tweezers (middle image) and a freely suspended membrane burdened with ~3 g of 2-propanol (lower image). (**d**) Higher magnification SEM image of a freely suspended membrane showing high contrast between membrane material and voids. The inset shows the edge of a membrane as viewed from the side. Scale bar: 200 nm (**e**) Higher magnification SEM micrograph showing a freely suspended perforated nanomembrane in tilted view. The background in the upper right corner visible in black and perforations appearing similarly dark are indicated by the arrows. (**f**) AFM image of the edge of a membrane on top of a silicon wafer. g, Height profile extracted from f corresponding to the white line.

**Figure 3:**
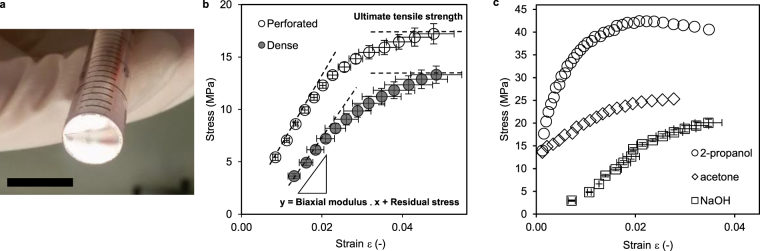
Mechanical properties with different solvents. (**a**) Photograph of a perforated nanomembrane suspended over the opening of a 9.4 mm diameter plastic tube used for bulging experiments. The nanomembrane is only visible due to reflection. Scale bar: 1 cm. (**b**) Stress - strain correlations of perforated and dense nanomembranes of identical matrix composition determined in aqueous environment. Material parameters extracted from the correlation are indicated. Dashed lines are a guide to the eye. (**c**) Stress - strain plots of perforated nanomembranes determined with the indicated organic solvent. Water was used for the nanomembranes incubated with 1 M NaOH beforehand.

**Figure 4:**
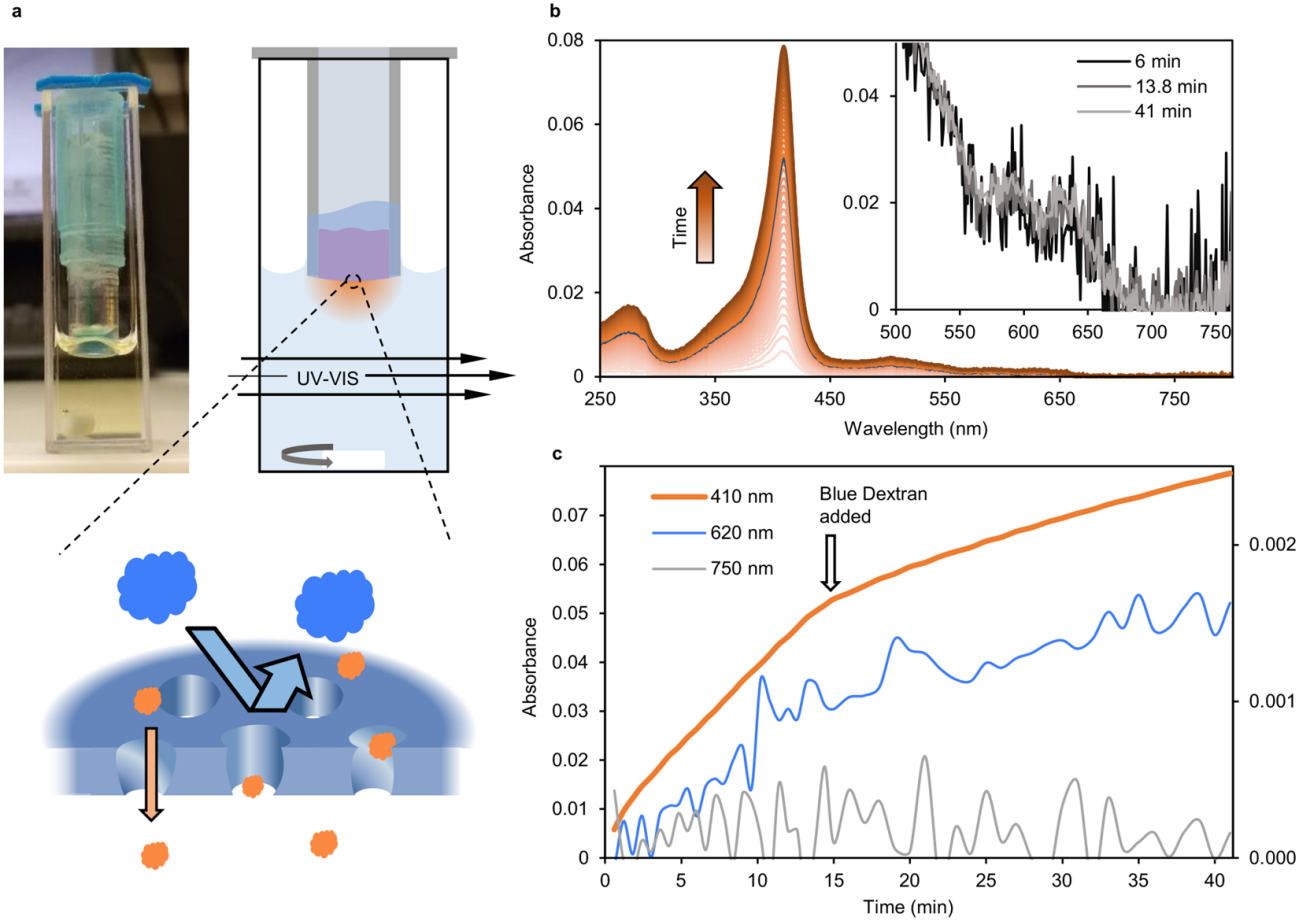
Diffusion based permeability assay. (**a**) Photograph and schematic representation of the assembly that is used in a spectrophotometer to follow size selective protein transport across perforated nanomembranes in real-time. The nanomembrane is freely suspended over the opening of the plastic tube (7 mm diameter) to separate the retentate solution (inside the tube) and the stirred permeate solution (inside the 10 mm pathlength cuvette). (**b**) UV-Vis absorbance spectra of the permeate solution over time (from light to dark orange). The highlighted spectrum was recorded directly before addition of Blue Dextran solution to the retentate side. The inset shows absorbance spectra normalized to unity at 410 nm to illustrate the absence of Blue Dextran based on their invariant shape. (**c**) Absorbance of the permeate solution at selected representative wavelengths: 410 nm for myoglobin, 620 nm for Blue Dextran and 750 nm for TiO_2_ nanoparticles. 620 nm and 750 nm values are scaled according to the secondary y-axis.

**Figure 5:**
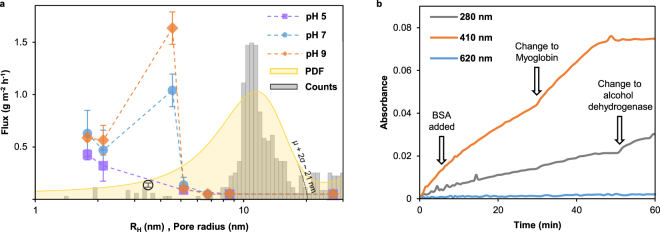
Separation performance for differently sized proteins and pore size distribution. (**a**) Transport rates of differently sized proteins across perforated nanomembranes plotted versus their hydrodynamic radius. The black circle represents bovine serum albumin at a smaller reported R_H_ value^45^. Grey bars represent the pore radius probability of occurrence while the corresponding probability density function is indicated in yellow as calculated from SEM images. The dashed line intersects the abscissa at ~21 nm and is indicative of µ + 2σ. b, Absorbance of permeate chamber at selected wavelengths during a diffusion experiment with cytochrome c where highly concentrated BSA has been added after 6 minutes. After 30 and 50 minutes the retentate solution was changed to myoglobin and alcohol dehydrogenase, respectively.

Ultrathin polymer films were produced by spin coating a ternary polymer mixture of PCGF, PEI and poly(D,L-lactide-*co*-glycolide) (PLGA) dissolved in chloroform onto a water-soluble polystyrene sulfonate sacrificial layer (Fig. 1a-1,a-2). Phase separation occurring during casting, governed by the different solubilities of the polymers in the common solvent, leads to small circular domains of PLGA that are dispersed in a continuous epoxy matrix (PCGF/PEI) (Fig. 1b). Covalent crosslinking of the functional residues within the continuous matrix, initiated at elevated temperature (Fig. 1a-3), results in high mechanical and chemical stability and pins this non-equilibrium morphology. Consequently, the epoxy matrix is rendered insoluble such that the PLGA domains can subsequently be dissolved selectively with the casting solvent to form regular perforations (Fig. 1a-4). Immersion into water dissolves the sacrificial layer to separate the perforated nanomembrane from the wafer. By appropriate immersion, water can penetrate and dissolve the sacrificial layer before the nanomembrane is submerged which leads to floating of the nanomembrane on the surface (Fig. 1a-5). In this state the nanomembranes maintain a stretched and completely flat shape rendering pick up procedures onto supports and tubes very easy (Fig. 2c). In contrast, when submerged, nanomembranes are freely floating and show a crumpled morphology. Note that the adhesive properties of the membrane material mediate tight attachment to a variety of materials for characterization so that clamping or gluing steps can be avoided (Fig. 2c). The entire fabrication process is typically completed within 20–30 minutes.

It is conceivable from previous studies^27,28^ that careful and adequate adjustment of polymer blend concentrations and spin coating parameters can lead to morphologies that are of proper scale for protein separations. Accordingly, the presented nanomembrane fabrication procedure was adjusted to yield regular voids of appropriate size. Specifically, the amount of each of the three polymers in the casting solution was varied in small steps while the total polymer concentration, and thereby the resulting thickness, was kept constant. While the membrane precursor ratio (initially at unity) was very slightly adjusted towards PCGF resulting in an increased stability, the ratio of thermoset resin to porogen was increased to 15:1 from the initially used 10:1. Thereby, the resulting dimensions of approximately 25 nm diameter (see Supplementary Figure 9 for pore radius distributions) of the porogen phases that penetrate the entire thickness (perforations) as shown in electron microscopy images (Fig. 2a,b,d and e) were obtained. These domains of phase separated PLGA have been dissolved to form regular perforations. The membranes shown in Fig. 2e,d were freely suspended during imaging to enhance the contrast between membrane material and voids to highlight the perforations. The inset in Fig. 2d shows a side view of the ripped edge of a membrane illustrating the low thickness and membrane spanning pores. A thickness of 68 nm was estimated from the membrane folds in Fig. 2a. Surface depressions of around 150 nm diameter and 20 nm depth originate from discontinuous domains of larger length scale which phase separate in parallel but do not penetrate the entire thickness of the nanomembrane matrix (Fig. 2f,g). This is in agreement with observations of Walheim and co-workers and the anticipated morphologies could be reproduced in the present study with different polymers. The crack like structures observed in SEM, best visible in Fig. 2b,d, are features not intrinsic to the nanomembrane surface but result from the poor wetting of the surface with sputter coated gold (Supplementary Figure 1)^31^. Line profiles extracted from atomic force microscopy (AFM) data confirm the nanoscale thickness (~75 nm) of the perforated polymer films. The dimensions and geometry of the cantilever tip obstructs adequate profiling since the small pore diameter does not allow sufficient penetration (Fig. 2g). The good correlation between UV absorbance of the nanomembranes with their chemical composition and thickness enables rapid determination of membrane thickness in a spectrophotometer (Supplementary Figure 2). Thereby, sufficiently robust perforated nanomembranes were determined to be typically 70 nm thick which is in good agreement with estimations from SEM and AFM.

## Mechanical and Chemical Properties

It was of great interest to verify whether addition of a third polymer and the introduction of perforations has considerable impact on the outstanding mechanical properties of the thermoset resin. Mechanical properties were determined by bulging tests with a specialized but simple setup. The nanomembranes were freely suspended over the opening of a tube (Fig. 3a) and burdened by pipetting increasing amounts of liquid onto the surface within the tube until membrane rupture. The resulting deflection of the nanomembranes was monitored by taking optical images and can be used to calculate stress and strain (Supplementary Section 1 and Fig. 3). The biaxial modulus, ultimate tensile strength and residual stress were derived (Table 1) from the stress (σ) – strain (ε) relationships obtained with different liquids (Fig. 3b,c). In contrast to air pressure the use of liquids allows the determination of mechanical stability in aqueous environment (under operating conditions), in hydrated or swollen state caused by other solvents. Perforated nanomembranes show similar mechanical stability as their non-perforated counterparts described and measured by Watanabe and Kunitake (Young’s modulus: 350 MPa equivalent to 500 MPa biaxial modulus^32^, ultimate tensile strength: 22 MPa). Table 1 lists the biaxial moduli (Y) determined for dense and perforated nanomembranes with our setup in wet state. They were in the same range with 506 MPa for dense and 700 MPa for perforated nanomembranes, respectively. Membrane rupture points, representing the ultimate tensile strength, were also similar with 14.8 and 17.9 MPa, respectively.

**Table 1:** Mechanical parameters determined from bulging tests.

	Y	UTS	σ_0_
	(Mpa)		
Perforated	700 ± 121	17.9 ± 1.7	0.5 ± 0.9
Dense	506 ± 74	14.8 ± 2.0	−2.4 ± 0.5
1 M NaOH	938 ± 166	20.8 ± 0.7	−3.8 ± 1.2
2-propanol	3242 ± 645	35.2 ± 7.2	8.7 ± 4.6
Acetone	959 ± 99	21.5 ± 5.1	13.7 ± 8.3

We have preferred bulging to the frequently used Strain-Induced Elastic Buckling Instability for Mechanical Measurement (SIEBIMM) since it is simpler to conduct and additional parameters (ultimate tensile strength and residual stress) can be derived. Nevertheless, residual stress is highly dependent on slight variations in the process of membrane pick up and should be neglected as a material related parameter. Studies on porous or perforated freestanding thin films rarely focus on mechanical strength although it is a potentially crucial parameter and Young’s modulus alone is not sufficient to adequately describe the mechanical robustness of a material. High mechanical strength of ultrathin separation layers will allow the use of supporting materials with larger pores ultimately decreasing overall mass transfer resistance. The presented bulging experiments reveal that the membranes exhibit high mechanical stability in spite of possessing only a moderate biaxial modulus compared to other polymeric perforated membranes^33^.

The nanomembranes are stable under harsh chemical conditions such as incubation with 1 M sodium hydroxide (pH = 14) for 30 minutes and in various organic solvents such as acetone and 2-propanol (Fig. 3c and Table 1). Their resistance towards sodium hydroxide is ideal for biotechnological applications, where materials are frequently cleaned and sanitized at high pH. The stability in organic solvents will allow a careful washing of the membranes to minimize amounts of leachables and extractables. Distinct changes in bulging behavior for acetone and 2-propanol manifested in high residual stress and considerable slack, respectively. Interestingly the membranes exhibited significantly higher strength and stiffness when bulged with 2-propanol. This might indicate that the nanomembrane matrix is not as easily penetrated by 2-propanol than by water or acetone since elastic properties scale negatively with swelling^34^.

Surface free energies (SFE) of both perforated as well as dense nanomembranes were estimated by contact angle (θ) measurements with several liquids of different polarity and surface tension according to Zisman^35^ and van Oss^36^, respectively (Supplementary Section 2). SFE values obtained by the method of Zisman were very similar for both dense and perforated nanomembranes (both ~26.5 mJ m^−2^) which is two orders of magnitude lower than that of silicon^37^ and in the area of polymer SFE promoting low protein adsorption^16^. Interestingly, the surface that forms an interface with air during casting (top surface) is less hydrophilic than the surface facing the sacrificial layer (lower surface). The wettability (cos (θ)) of the lower surface (0.97) with water is optimal for low adsorption^38^ but the overall wetting behavior led to inconclusive results in the framework of Zisman (Supplementary Figure 7). The top surface wettability (0.2) is in the moderate hydrophilic range where many membrane and chromatography materials reside (Supplementary Table 1). When evaluated according to van Oss the top surface showed low values for the polar components with a more pronounced positive component (Positive: 2.3 mJ m^−2^ and Negative: 1.8 mJ m^−2^) and a disperse component corresponding well to the Zisman value (32.0 mJ m^−2^). The lower surface showed increased polar components with a similarly pronounced positive component (Positive: 22.6 mJ m^−2^ and Negative: 12.9 mJ m^−2^) alongside a very similar disperse component (32.4 mJ m^−2^). It has been shown that surfaces with high polar components tend to adsorb proteins more tightly and moderate values of SFE of both components are advantageous and have been linked to low protein adhesion^16^. Moreover, the asymmetric wetting behavior is an intriguing feature which may open up venues for functionalization, ordered and directed immobilization of functional enzymes.

## Separation Performance

We have characterized the transport of proteins through the perforated nanomembranes by diffusion with a spectrophotometric setup. Separation performance has rarely been determined for freestanding nanomembranes and generally on µm scale whereas we demonstrate this at 1000-fold larger scale (~40 mm²). The respective nanomembrane is freely suspended over the circular opening of a plastic tube and the inside of the tube (the retentate chamber) is partially filled with the test solution. The end of the tube that is sealed by the nanomembrane is inserted into a quartz cuvette filled with buffer which acts as the permeate chamber that is constantly stirred by a magnetic bar and directly monitored for UV-Vis absorbance (Fig. 4a). The specific absorbance of the analytes is used to independently monitor their concentration on the permeate side in real time (Absorbance spectra see Supplementary Figure 4). It can be confirmed that the membrane is defect-free by proving the rejection of large probes such as Blue Dextran (2000 kDa, 50 nm diameter) or TiO_2_ nanoparticles (100 nm diameter). Figure 4b,c summarizes a single separation experiment for illustration purposes where a solution initially containing myoglobin and TiO_2_ nanoparticles has been loaded into the retentate chamber. Figure 4b shows the UV-VIS absorbance of the permeate solution over time. The absence of changes in absorbance at wavelengths over 750 nm in the permeate chamber indicates that the 100 nm nanoparticles have been fully retained. After 15 min a solution of Blue Dextran, which has a broad absorbance peak at 625 nm, has been thoroughly admixed to the retentate with a pipette. A minimal increase in permeate absorbance at 625 nm over time (Fig. 4c) can be attributed to myoglobin rather than Blue Dextran. The correlation of the rates of change in absorbance at 410 nm and 625 nm is present before as well as after Blue Dextran addition. Moreover, the spectra shown in the inset of Fig. 4b were normalized to unity at the peak maximum of myoglobin (410 nm) to clearly visualize that the shapes of the spectra did not change upon addition of Blue Dextran, i.e. no emerging absorbance peak around 625 nm is observable. Therefore a contribution of Blue Dextran to the spectral absorbance can be precluded corroborating that the membrane was intact. Note the distinct decrease in myoglobin diffusion rate upon addition of the Blue Dextran solution as a consequence of dilution (Fig. 4c). With such experiments, it can be clearly demonstrated that proteins pass through the perforated nanomembranes while larger species are fully retained. With non-perforated membranes there was no observable change in spectral absorbance at any wavelength (Supplementary Figure 5). It is also worth noting that the freely suspended nanomembranes are robust enough to withstand the perturbations caused by retentate mixing with a pipette, the changing of retentate as well as transfer to a different permeate chamber cuvette.

## Permeability of Various Model Compounds

From the time-dependent increase of permeate absorbance the diffusion or transport rate across the membrane was derived. Since the concentration gradient changes over time and the retentate side is far from being ideally mixed, the initial slope was considered to derive representative values for the transport rate. We have determined the transport rates for different model proteins of increasing molecular weight and hydrodynamic size (Table 2).

In each diffusion experiment the structural integrity of the respective nanomembrane was confirmed by full rejection of the large probe molecule Blue Dextran and each membrane was tested with several (at least two) different protein solutions to put the observed rates into context. We determined protein diffusion rates at pH values 5, 7 and 9 and examined the influence on separation behavior. Diffusion rates are average values of at least 3 different membranes and summarized in Table 2 and plotted versus the respective hydrodynamic radius in Fig. 5a. Additionally, a histogram of the pore radius distribution (grey bars) as estimated from SEM images (Supplementary Figures 8 and 9) is provided in Fig. 5a. The probability density function of the pore radius is overlaid in yellow which has its peak maximum at roughly 12 nm and 95% are smaller than 21 nm. As expected for nanomembranes with defined pore geometry a sharp transport rate cut-off is observable which is located between alcohol dehydrogenase and apoferritin. Diffusion of apoferritin, thyroglobulin and Blue Dextran was not observed in any experiment where an intact nanomembrane was confirmed. However, we observed changes of permeate spectra over time presumably caused by impurities which were present in apoferritin solutions despite dialysis with commercial devices (Supplementary Figure 6). Diffusion rates determined for alcohol dehydrogenase were significantly higher than those of myoglobin and cytochrome *c* at pH 7 and 9. A more negatively charged surface might be the reason for this behavior which adds an additional mode of selectivity^17^. The surface free energy calculations point towards positively charged surfaces of the nanomembranes which is in agreement with the general trend of lower transport rates as the proteins get more positively charged with decreasing pH. Interestingly, we observed very low diffusion rates for bovine serum albumin although its nominal molecular mass is less than half as that of alcohol dehydrogenase and despite an overall negative charge^39^. To preclude that this observation was due to BSA adsorption to the pore walls it is adequate to refer to the experiment illustrated in Fig. 5b. After initial exclusive diffusion of cytochrome c (~0.57 g m^−2^ h^−1^) for 6 minutes a small amount of highly concentrated (50 g L^−1^) BSA solution was added to a final concentration of 5 g L^−1^ to the retentate whereby cytochrome c concentration is only marginally changed. It is apparent that the diffusion rate of cytochrome c was not significantly altered by the addition of BSA solution. Later the retentate solution was changed altogether twice, first after 30 minutes to 1 g L^−1^ myoglobin (~0.45 g m^−2^ h^−1^) and then after 50 minutes to 1 g L^−1^ alcohol dehydrogenase (~1.1 g m^−2^ h^−1^) showing that these diffusion rates were also largely unaffected. Therefore we conclude this might be attributed to the proposed non-globular conformation of BSA with dimensions of 14 nm × 4 nm × 4 nm^40^. Many different sizes have been reported for different conformations of bovine serum albumin most of which are larger than that of alcohol dehydrogenase^39,41,42^. Moreover, the observed transport rates nicely reflect a general trend of larger diameters at pH values distant from neutral pH highlighting the high-resolution size selectivity. Complete rejection of proteins larger than 13 nm in hydrodynamic diameter shows that the perforations are of appropriate scale to be used in protein separations. By carefully adjusting parameters such as polymer concentrations, porogen molecular weight and spin coating rotation speed the scale of the resulting nanofeatures and thereby the separation behavior will be readily tunable.

**Table 2:** Characteristics of the proteins used to characterize permeability cut-off and observed transport rates.

Analyte	Size		Charge				Transport rate		
	MW	R_H_^a^	IEP^b^	charge at pH			(g m^−2^ h^−1^)		
	(kDa)	(nm)	(pH)	5	7	9	pH 5	pH 7	pH 9
Cytochrome c	12.5	1.78	10.04	+	+	+/0	0.38	0.58	0.54
Myoglobin	16.7	2.12	6.85/7.33	+	+/0	—	0.27	0.42	0.51
Bovine serum albumin	66	5.15	4.9	0	—	—	0.04	0.09	0.05
Alcohol dehydrogenase	150	4.55	5.4–5.8	+/0	—	—	n.d.	0.99	1.59
Apoferritin	443	6.73	4	—	—	—	n.d.	0	0
Thyroglobulin	670	8.58	4.5–5.0	0/—	—	—	0	0	0
Blue Dextran	2000	26.89	7	+	0	—	0	0	0

n.d.: 1 g L^−1^ protein solutions were not used for diffusion experiments due to aggregation.

^a,b^ Values for the hydrodynamic radii (R_H_) and the isoelectric points (IEP) were taken from the literature as listed in Supplementary Table 2.

## Conclusions

We have developed a procedure to fabricate ultrathin hydrophilic mesoporous polymer based membranes with characteristics suitable for protein separations. Our focus was on defined pore geometry of adequate size and biocompatibility as opposed to membranes with tortuous paths and inorganic or hydrophobic membranes. The demonstrated nanomembranes can be freely suspended over tens of mm² during characterization, highlighting their outstanding mechanical properties. They preserve their robustness in organic solvents and show resistance to high pH values which is ideal for implementation in biotechnological applications. Furthermore, the hydrophilic nature and low surface free energy with moderate polar components are optimal for low protein adsorption. The observed sharp cut-off between permeation and retention of proteins is in the desired range where industrially important biomolecules reside emphasizing the possibility for group separations. Transfer to any kind of desired support material is enabled by their freestanding nature. The membranes’ asymmetric wetting behavior might open venues for directed immobilization and functionalization with biomolecules making them interesting for research. The development and demonstration of this fast and simple fabrication procedure is intended to reinforce and advance the economic feasibility of nanomembranes and thereby shrink the gap towards industrial applicability. We suggest that future development concerning such polymer-based nanomembranes should be dedicated to further improve mechanical stability and porosity of adjustable size distribution.

## Methods

### Materials

All chemicals, proteins and solvents were purchased from Sigma-Aldrich unless otherwise mentioned. HQ-H_2_O (0.055 µS cm^−1^) was used throughout the whole study. The chemicals Poly(sodium 4-styrenesulfonate) (PSS, Mw = 70 000), Poly[(*o*-cresyl glycidyl ether)-*co*-formaldehyde] (PCGF, Mn = 870), branched Polyethylenimine (PEI, Mn = 10 000) and Poly(D,L-lactide-*co*-glycolide) (PLGA, lactide:glycolide 75:25, Mw 76 000–115 000) were used as received. The polymers PCGF and PEI were dissolved in chloroform (CHCl_3_) to a concentration of 15 mg mL^−1^ and PLGA to a concentration of 2 mg mL^−1^. Chloroform solutions were filtered with 0.22 µm PTFE syringe filters. These solutions can be stored for months in glass vials with PTFE lined screw caps.

Test liquids for contact angle measurements diiodomethane (Sigma-Aldrich, 158429), ethylene glycol (Merck, 109621), formamide (Roth, P040.1), glycerol (Fisher Scientific, BP229-1) and toluene (Sigma-Aldrich, 648566) were used as received.

Protein formulations, except apoferritin, were used as received to prepare solutions with a 1 g L^−1^ protein concentration. Blue Dextran solutions were prepared to a concentration of 10 g L^−1^. Protein and Blue Dextran solutions were buffered with a solution of desired pH prepared according to Britton and Robinson^43^ with buffering species concentrations of 0.04 M and additional 0.1 M NaCl. Apoferritin was dialyzed (received as a 20 g L^−1^ solution) with a 2000-fold excess buffer volume with pH = 7.0 in a dialysis cassette (Slide-A-Lyzer™ 10 K MWCO, Thermo Fisher) for 24 hours from which 1 g L^−1^ apoferritin solutions were prepared with desired pH.

Titanium dioxide nanoparticles (TiO_2_-NP) were obtained from CINKARNA (Celje) and a 2 g L^−1^ suspension was prepared with buffer of pH = 9 as described above. To counteract aggregation and settling, TiO_2_-NP were treated in an ultrasonication bath for 30 minutes prior to use.

### Nanomembrane fabrication

Silicon wafers with one polished side and 50.8 mm (2 inch) diameter (SiMat) were immersed in concentrated (96%) sulfuric acid for at least 15 minutes for cleaning and then thoroughly rinsed with distilled water prior to spin coating. All spin coating steps were done with a WS650Mz-23NPP Spincoater (Laurell). Sacrificial layers were formed by spin coating 300 µL of a 5% w/v solution of PSS in HQ-H_2_O onto a cleaned silicon wafer at 3000 rpm for one minute. For the preparation of perforated nanomembranes 110 µL of PCGF and 90 µL of PEI solution (15 mg mL^−1^ each) were mixed at room temperature for 5 minutes before 100 µL of PLGA (2 mg mL^−1^) solution was added. This membrane casting solution was vigorously shaken for one minute before 150 µL were spin coated on top of a freshly prepared sacrificial layer at 5000 rpm for one minute. The thermosetting resin was then cured by placing the silicon wafer onto an 80 °C hot plate for 20 minutes. Dense nanomembranes were produced with the same procedure but instead using 100 µL of both PCGF and PEI, without addition of PLGA and without the following 1 minute of vigorous stirring.

### Selective solvent etching (SSE)

The wafer bearing the cured nanomembrane on top of the sacrificial layer was allowed to cool to room temperature. The PLGA pore templates were subsequently dissolved by immersing the silicon wafer in chloroform for one minute. The wafer was then allowed to dry in air before the, as of now perforated, nanomembrane was rinsed twice with CHCl_3_.

### Nanomembrane release

The nanomembrane could then be released on the surface of distilled water by gradually immersing the silicon wafer at an angle of approximately 30° thereby dissolving the sandwiched sacrificial layer. The floating membrane could then be picked up onto any desired substrate or device for further investigations.

### Bulging

For bulging tests the individual floating nanomembrane pieces were picked up with a plastic tube of 9.7 mm diameter so that one opening of the tube is completely sealed. The nanomembranes adhered tightly to the tube walls without the need for any further fixation or clamping and were brought into air for drying. The tube was then vertically aligned with the sealed end facing downwards whereby the nanomembranes could then be gradually pressurized with increasing amounts of test liquid contained by the tube. The resulting deflection of the nanomembranes was captured by a digital camera (791 × 588 pixels, resolution: 265 pixels mm^−1^) in steps of 100 µL and correlated to the hydrostatic pressures. Stress and strain calculation was done as described in the Supplementary section 1. To test the resistance to alkaline environment nanomembranes were floated on 1 M NaOH solution for 30 minutes before they were neutralized on the surface of water for 30 minutes. Following regeneration they were picked up for bulging with water as a pressurizing liquid. When bulged with organic solvents, nanomembranes were not dissolved but stable and only ruptured by means of increasing pressure after several minutes.

### Diffusion

Nanomembranes were picked up on a 7 mm diameter plastic tube to seal one opening completely (Fig. 4a). Unless otherwise stated, 50 µL of filtrate solution (prepared from 45 µL of protein solution plus 5 µL of Blue Dextran solution) were then pipetted into the tube and on top of the nanomembrane. The sealed end of the tube was then immersed into 980 µL of permeate solution (Buffer without protein or Blue Dextran) contained in a 1 cm path length cuvette. The permeate solution volume of 980 µL was chosen so that the membrane edges were not immersed to ensure that the permeating species have to cross the membrane in order to enter the permeate side. UV-VIS spectra (230–800 nm) of the permeate solution were recorded at least every minute with a Cary 60 UV-Vis Spectrophotometer. The filtrate side was repeatedly mixed with a pipette and the permeate side was permanently stirred with a magnetic stirrer.

### Contact angle measurements

Contact angles (θ) of the test liquids diiodomethane, ethyleneglycol, formamide, glycerol, water and toluene with the respective surface were determined in air. Optical images were taken with a DSA30 contact angle goniometer (Krüss) and the average value of both measureable θ in each photograph was used for further calculations. Contact angles were allowed to reach a constant value for 5 to 30 seconds after drop deposition and θ were obtained from at least 3 individual droplets of 0.5–1 µL. Surface free energy calculations were done as described in Supplementary section 2 and Supplementary Figure 7.

### High resolution imaging

For high resolution imaging the nanomembranes were picked onto small pieces of highly polished silicon wafers (see above and Fig. 2c) or on copper grids for imaging in freely suspended state. Samples for scanning electron microscopy (SEM) were sputter coated with a thin conductive layer of gold and contacted with the SEM-stubs with conductive silver paste. SEM images were obtained with a QuantaTM 250 field-emission environmental scanning electron microscope with a Schottky field emission gun (FEI Europe B.V.) operated at 20 kV in a high vacuum of 1 × 10^−6^ mbar. Atomic force microscopy (AFM) images were obtained with a Nanowizard II atomic force microscope (JPK Instruments) using SiN cantilevers DNP-S10 (Bruker) with a nominal spring constant of 0.12 N m^−1^ having pyramidal tips with a nominal radius of 10 nm. Scans were done in contact mode in air. The probed areas were typically scanned with a 512-line resolution and a scan frequency of 2 Hz.

### Pore size distribution

SEM images were processed with Mathematica 11.0 (Wolfram Research)^44^. A combination of several filters was applied to the respective SEM image in the following order: Blur, Erosion, RidgeFilter, MorphologicalComponents. Components too large to be of interest (background or artifacts) were deselected. The contours of the remaining components were adjusted to fit those in the image as judged by eye. This was done by detection of the component-edges (EdgeDetect) which were then thickened by a Dilation filter depleting the encompassed area to fit the pore area. The resulting image was inverted to give the encompassed areas (and the non-porous area which was deselected). For the obtained components, the “EquivalentDiskRadius” was calculated giving the pore radii. A series of images from several intermediate processing steps of a selected SEM image is shown in Supplementary Figure 8.

### Data availability

The datasets generated during and/or analyzed during the current study are available from the corresponding author on reasonable request.

## Electronic supplementary material

Supplementary Information

## References

[1] Saraswat M (2013). Preparative Purification of Recombinant Proteins: Current Status and Future Trends. BioMed Res. Int..

[2] Saxena A, Tripathi BP, Kumar M, Shahi VK (2009). Membrane-based techniques for the separation and purification of proteins: An overview. Adv. Colloid Interface Sci..

[3] Snyder JL (2011). An experimental and theoretical analysis of molecular separations by diffusion through ultrathin nanoporous membranes. J. Membr. Sci..

[4] Mireles, M. & Gaborski, T. R. Fabrication techniques enabling ultrathin nanostructured membranes for separations. *ELECTROPHORESIS* n/a-n/a 10.1002/elps.201700114. 10.1002/elps.201700114 PMC5909070 28524241

[5] Gottschalk, U. Disposables in Downstream Processing. in *Disposable Bioreactors* 171–183 (Springer, Berlin, Heidelberg, 2009). 10.1007/10_2008_22.

[6] Chico, K. Z. J. M. P.-C. E. The Impact of Disposables on Project Economics in a New Antibody Plant: A Case Study. Available at: http://www.biopharminternational.com/impact-disposables-project-economics-new-antibody-plant-case-study. (Accessed: 4th September 2017).

[7] Striemer CC, Gaborski TR, McGrath JL, Fauchet PM (2007). Charge- and size-based separation of macromolecules using ultrathin silicon membranes. Nature.

[8] Tong HD (2004). Silicon Nitride Nanosieve Membrane. Nano Lett..

[9] Lin X, Yang Q, Ding L, Su B (2015). Ultrathin Silica Membranes with Highly Ordered and Perpendicular Nanochannels for Precise and Fast Molecular Separation. ACS Nano.

[10] Klein MJK (2011). SiN membranes with submicrometer hole arrays patterned by wafer-scale nanosphere lithography. J. Vac. Sci. Technol. B Nanotechnol. Microelectron. Mater. Process. Meas. Phenom..

[11] Lipson AL, Comstock DJ, Hersam MC (2009). Nanoporous Templates and Membranes Formed by Nanosphere Lithography and Aluminum Anodization. Small.

[12] Montagne F, Blondiaux N, Bojko A, Pugin R (2012). Molecular transport through nanoporous silicon nitride membranes produced from self-assembling block copolymers. Nanoscale.

[13] Nuxoll EE, Hillmyer MA, Wang R, Leighton C, Siegel RA (2009). Composite Block Polymer−Microfabricated Silicon Nanoporous Membrane. ACS Appl. Mater. Interfaces.

[14] Park HJ, Kang M-G, Guo LJ (2009). Large Area High Density Sub-20 nm SiO2 Nanostructures Fabricated by Block Copolymer Template for Nanoimprint Lithography. ACS Nano.

[15] Wahlgren M, Arnebrant T (1991). Protein adsorption to solid surfaces. Trends Biotechnol..

[16] Frank BP, Belfort G (2001). Atomic Force Microscopy for Low-Adhesion Surfaces:  Thermodynamic Criteria, Critical Surface Tension, and Intermolecular Forces. Langmuir.

[17] Qiu X (2013). Selective Separation of Similarly Sized Proteins with Tunable Nanoporous Block Copolymer Membranes. ACS Nano.

[18] Yang SY (2008). Virus Filtration Membranes Prepared from Nanoporous Block Copolymers with Good Dimensional Stability under High Pressures and Excellent Solvent Resistance. Adv. Funct. Mater..

[19] Vriezekolk EJ, Kudernac T, de Vos WM, Nijmeijer K (2015). Composite ultrafiltration membranes with tunable properties based on a self-assembling block copolymer/homopolymer system. J. Polym. Sci. Part B Polym. Phys..

[20] Yu H (2015). Self-Assembled Asymmetric Block Copolymer Membranes: Bridging the Gap from Ultra- to Nanofiltration. Angew. Chem. Int. Ed..

[21] Yang SY (2006). Nanoporous Membranes with Ultrahigh Selectivity and Flux for the Filtration of Viruses. Adv. Mater..

[22] Kang C (2015). Ultrathin, freestanding, stimuli-responsive, porous membranes from polymer hydrogel-brushes. Nanoscale.

[23] Watanabe H, Kunitake T (2007). A Large, Freestanding, 20 nm Thick Nanomembrane Based on an Epoxy Resin. Adv. Mater..

[24] Watanabe H, Muto E, Ohzono T, Nakao A, Kunitake T (2009). Giant nanomembrane of covalently-hybridized epoxy resin and silica. J. Mater. Chem..

[25] Andersson MM, Hatti-Kaul R (1999). Protein stabilising effect of polyethyleneimine. J. Biotechnol..

[26] Kunitake T (2016). Perspectives: Synthetic Bilayer Membrane and Giant Nanomembrane. Langmuir.

[27] Nauman EB, He DQ (1994). Morphology predictions for ternary polymer blends undergoing spinodal decomposition. Polymer.

[28] Walheim S, Ramstein M, Steiner U (1999). Morphologies in Ternary Polymer Blends after Spin-Coating. Langmuir.

[29] Puiggalí-Jou A, Medina J, del Valle LJ, Alemán C (2016). Nanoperforations in poly(lactic acid) free-standing nanomembranes to promote interactions with cell filopodia. Eur. Polym. J..

[30] Puiggalí-Jou, A. *et al*. Confinement of a β-barrel protein in nanoperforated free-standing nanomembranes for ion transport. *Nanoscale*10.1039/C6NR04948F (2016). 10.1039/c6nr04948f 27714137

[31] Ruffino F, Torrisi V, Marletta G, Grimaldi MG (2011). Growth morphology of nanoscale sputter-deposited Au films on amorphous soft polymeric substrates. Appl. Phys. A.

[32] Small MK, Nix WD (1992). Analysis of the accuracy of the bulge test in determining the mechanical properties of thin films. J. Mater. Res..

[33] Zimnitsky D, Shevchenko VV, Tsukruk VV (2008). Perforated, Freely Suspended Layer-by-Layer Nanoscale Membranes. Langmuir.

[34] Candau, S., Bastide, J. & Delsanti, M. Structural, elastic, and dynamic properties of swollen polymer networks. in *PolymerNetworks* 27–71 (Springer, Berlin, Heidelberg, 1982). 10.1007/3-540-11471-8_2.

[35] ZISMAN, W. A. Relation of the Equilibrium Contact Angle to Liquid and Solid Constitution. in *Contact Angle*, *Wettability*, *and Adhesion* **43**, 1–51 (AMERICAN CHEMICAL SOCIETY, 1964).

[36] Good, R. J. & Oss, C. J. van. The Modern Theory of Contact Angles and the Hydrogen Bond Components of Surface Energies. in *Modern Approaches to Wettability*1–27 (Springer, Boston, MA, 1992). 10.1007/978-1-4899-1176-6_1.

[37] Hara S, Izumi S, Kumagai T, Sakai S (2005). Surface energy, stress and structure of well-relaxed amorphous silicon: A combination approach of ab initio and classical molecular dynamics. Surf. Sci..

[38] Sethuraman A, Han M, Kane RS, Belfort G (2004). Effect of Surface Wettability on the Adhesion of Proteins. Langmuir.

[39] Böhme U, Scheler U (2007). Effective charge of bovine serum albumin determined by electrophoresis NMR. Chem. Phys. Lett..

[40] Wright AK, Thompson MR (1975). Hydrodynamic structure of bovine serum albumin determined by transient electric birefringence. Biophys. J..

[41] Babcock JJ, Brancaleon L (2013). Bovine serum albumin oligomers in the E- and B-forms at low protein concentration and ionic strength. Int. J. Biol. Macromol..

[42] Ferrer ML, Duchowicz R, Carrasco B, de la Torre JG, Acuña AU (2001). The conformation of serum albumin in solution: a combined phosphorescence depolarization-hydrodynamic modeling study. Biophys. J..

[43] Britton HTS, Robinson RA (1931). CXCVIII.—Universal buffer solutions and the dissociation constant of veronal. J. Chem. Soc. Resumed.

[44] Wolfram Research, Inc., Mathematica, Version 11.1, Champaign, IL (2017).

[45] Axelsson I (1978). Characterization of proteins and other macromolecules by agarose gel chromatography. J. Chromatogr. A.

