# Supplementary material for: Freely suspended perforated polymer nanomembranes for protein separations
Source: Sci Rep. 2018 Mar 13;8:4410. doi: 10.1038/s41598-018-22200-4 (PMC5849607; doi:10.1038/s41598-018-22200-4)
Supplement: Supplementary file 1 — Supplementary Information [file 41598_2018_22200_MOESM1_ESM.pdf]

Freely suspended perforated polymer nanomembranes for protein separations

Christian Schuster[1], Agnes Rodler[1], Rupert Tscheliessnig[1], Alois Jungbauer[1,2]*

[1]Austrian Centre of Industrial Biotechnology, Vienna, Austria,

[2]University of Natural Resources and Life Sciences, Vienna, Austria

*e-mail: alois.jungbauer@boku.ac.at

1. Bulging tests - calculation of mechanical properties 2
2. Surface free energy by contact angles 4
3. SEM micrograph image processing for pore size distributions 5
4. Additional SEM images 6
5. Thickness estimation by UV Absorbance 7
6. Bulging test setup 9
7. UV-VIS Absorbance spectra of used analytes 10
8. Non – perforated nanomembrane diffusion experiment with myoglobin. 11
9. Apoferritin diffusion experiment 12
10. Zisman plot 13
11. Feature detection and pore size distribution from SEM images 14
12. Pore size distributions obtained from SEM images 15
13. Supplementary References 18

1. **Bulging tests - calculation of mechanical properties**

Mechanical properties were derived from plots of stress versus strain which were estimated as described later in the text. The biaxial moduli were calculated from the slope of the initial linear range of the stress-strain relationships (linear regression from at least 4 datapoints) and corrected according to Small and Nix[1] in the following manner:

$$Y = \frac{Y_{calc}}{1-0.24\nu-0.00027(1-\nu)\sigma_0} \quad \text{(Equation 1)}$$

where ν is Poisson's ratio (0.3 was assumed in all instances) and σ_0 is the residual stress which is given by the σ-intercept of the used linear regression. Furthermore, the relationship of biaxial- (Y) and elastic modulus (E) is given by:

$$Y = \frac{E}{1-\nu} \quad \text{Equation 2}$$

Stress (σ) induced by the hydrostatic pressure and the resulting deflection of the nanomembrane was calculated according to:

$$\sigma = \frac{Pa^2}{4hd} \quad \text{Equation 3}$$

with notation as indicated in Figure S 3-a. The hydrostatic pressure was calculated taking the inner cross-section of the tube as pressurized area which is burdened by the weight of the accumulating solvent with correction for the capillary pressure according to:

$$P = \frac{\rho g V}{A} - \Delta p_C \quad \text{Equation 4}$$

where ρ is the density of the liquid, g is the gravitational constant (9.81 m s^{-2}), V is the volume of the liquid and A is the inner cross-sectional area of the tube. The capillary pressure Δp_C was estimated according to:

$$\Delta p_C = \frac{2\gamma \cos\theta}{r} \quad \text{Equation 5}$$

where γ is the surface tension of the liquid, θ is the contact angle of the meniscus with the tube surface as indicated in Figure S 3-a and r is the inner radius of the tube.

Strain (ε) was estimated according to:

$$\varepsilon = \frac{2d^2}{3a^2} \quad \textit{Equation 6}$$

with notation as indicated in Figure S 3-a.

It is necessary to point out that the outer tube radius (a) was used as the geometry parameter for the pressurized spherical cap model whereas the inner tube radius (r) was used to derive the hydrostatic pressure. The membrane is indeed bulged over the outer diameter of the tube and the major part of the liquid column has radius r. However, the deviation of the liquid column from cylindrical form, as indicated in Figure S 3-b, leads to a slight overestimation of hydrostatic pressure as the liquid height will be lowered. The liquid volume contained by B (Figure S 3-b) is roughly 4 µL and the resulting underestimation of hydrostatic pressure was neglected.

2. Surface free energy by contact angles

The contact angle (θ) values determined for perforated nanomembranes (top and lower surface) and for dense nanomembranes with 6 different liquids were evaluated according to Zisman[2] and according to van Oss[3].

According to Zisman the surface free energy can be estimated by calculating the critical surface tension of an imaginary liquid that would ideally wet (contact angle of 0 °) the surface in question. Hence, when plotting the liquids surface tensions vs their cos (θ), extrapolation of this correlation to cos (θ) = 1 gives the surface free energy.

Van Oss splits the interfacial tensions into three components: disperse interactions (σ_D) and polar interactions of positive (σ_+) and negative nature (σ_-). Following the equation with L for liquid and S for surface:

$$\sigma_L\,(cos\,\theta + 1) = 2(\,\sqrt{\sigma_D^L\,\sigma_D^S} + \sqrt{\sigma_-^L\,\sigma_+^S} + \sqrt{\sigma_+^L\,\sigma_-^S}\,) \qquad \textit{Equation 7}$$

we solved the resulting matrix system with 6 liquids for each of the three probed surfaces. The equation:

$$a_i = M_{ij} b_j \qquad \textit{Equation 8}$$

was rearranged:

$$a_i M_{ij}^{-1} = b_j \qquad \textit{Equation 9}$$

to solve for b_j where M_{ij}^{-1} is the Moore-Penrose pseudoinverse of the rectangular matrix M_{ij}. Here a_i comprises $\sigma_L\,(\cos\theta + 1)$ of the liquid i, the matrix components of M_{ij} hold the surface tension components j of the liquid i and b_j holds the surface energies j of the sampled surface.

3. SEM micrograph image processing for pore size distributions

The semi-automated detection of circular features was performed with a Mathematica routine (see Figure S 8) for various different SEM micrographs and detected pores were evaluated for their size (see Figure S 9). For each individual image the parameters for the applied filters had to be adjusted in accordance with resolution, contrast, brightness and magnification. The outlines of the detected features were superimposed onto the original images (as in Figure S 8-f) to aid parameter adjustment by sound operator judgement. It is worth noting that the algorithms tend to neglect doubtful features since putative 'pores' weak in contrast are both, less likely to be detected and more probable to be no true perforations. On the contrary, putative pores rich in contrast (i.e. darker features) are more probable to be membrane spanning and are selected preferentially.

It is furthermore dependent on the resolution and magnification of the SEM micrographs whether features can be adequately detected, measured and classified on a nanometer scale. The images processed in this study to evaluate pore size distributions exhibited a physical pixel size of less than 2.5 nanometers. Thus, pixels that are falsely allocated to or neglected from the pore area account for less than ten percent error of the calculated radius for radii larger than 2.9 nm. This error is lower than one percent for radii larger than 10 nm.

4. Additional SEM images

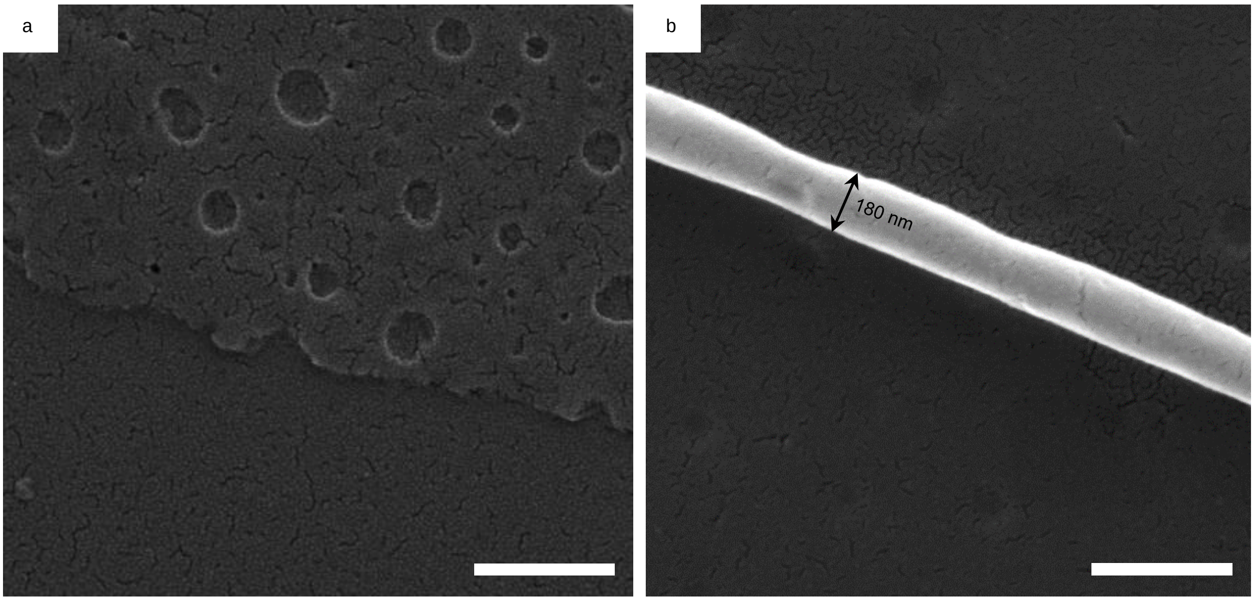

Supplementary Figure 1: Scanning electron microscopy images of nanomembranes on top of a silicon wafer.

a, A perforated nanomembrane on top of a highly polished silicon wafer. The presence of the small crack like structures on the wafer surface demonstrates that these are imperfections in the sputter-deposited gold coating. b, Image of a nanomembrane without perforations. The nanomembrane was cast from a solution with increased porogen (PLGA) concentration resulting in different phase separation phenomena. Note that the surface depressions of approximately 100 nm are still present but less pronounced. Scale bars are 500 nm.

5. Thickness estimation by UV Absorbance

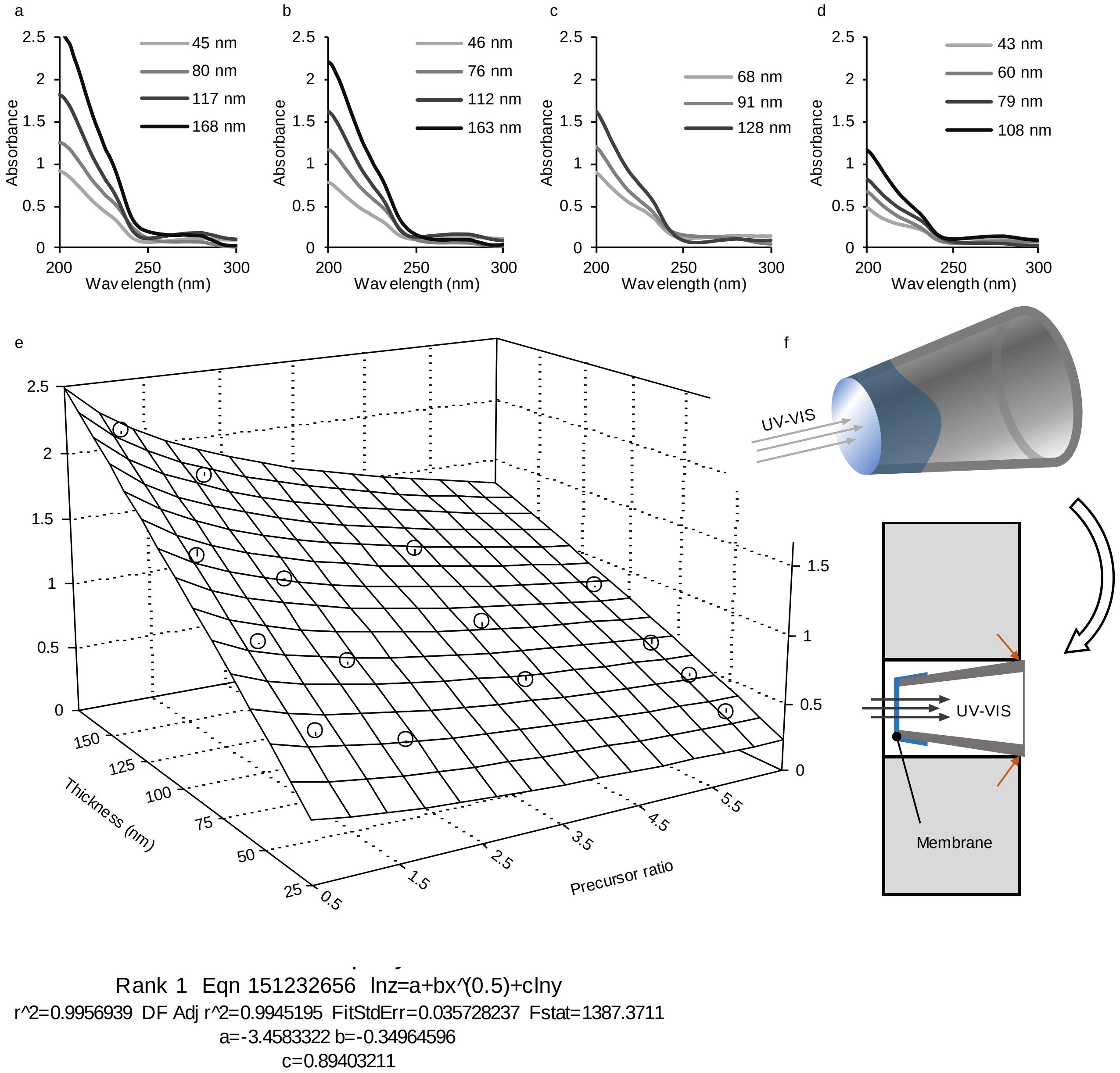

Supplementary Figure 2: Correlation of nanomembrane chemical composition, thickness and UV absorbance.

a – d, Absorbance spectra of nanomembranes with different precursor ratios (i.e. reactive group ratios in the form of amine:epoxy) of 1.1:1 (a), 2.0:1 (b), 4.1:1 (c) and 6.1:1 (d) and different thicknesses determined with atomic force microscopy height profiles. e, 3D-Plot of absorbance at 210 nm in dependence of thickness and chemical composition of the respective nanomembrane. The best surface fit was obtained by fitting the data to the equation ln(Absorbance)=a + b * Ratio$^{0.5}$+ c * ln (Thickness). Fitting was done with TableCurve3D v3. f, Schematic of the setup used to measure

the UV-VIS absorbance spectra in a spectrophotometer. The nanomembrane is freely suspended over the opening of a conical plastic tube, orange arrows indicate contact points of the tube with the specialized mounting cuvette.

6. Bulging test setup

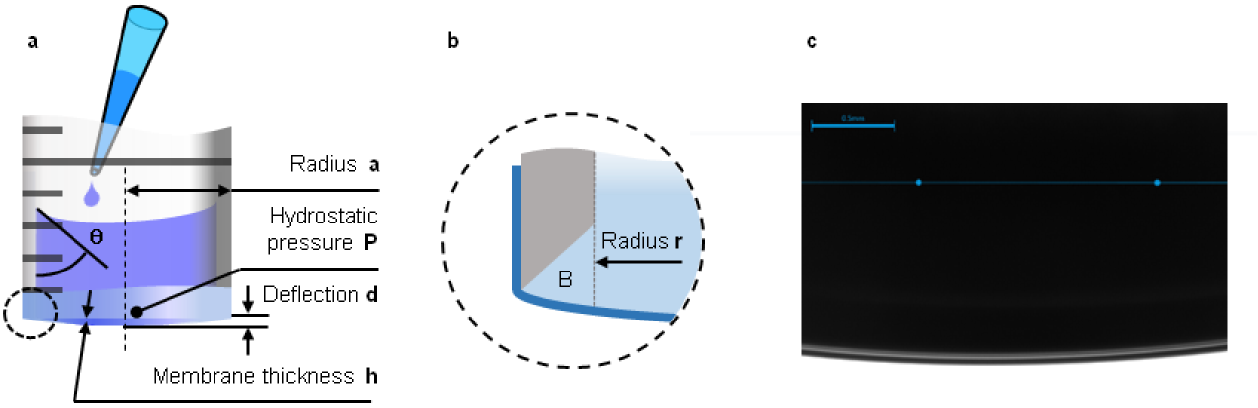

Supplementary Figure 3: Schematic setup and geometry of bulging tests.

a, b, Schematic representation of the bulging test setup (a) with indicated parameters necessary to derive stress – strain relationship (a and b) and a more detailed representation of the bulging tube geometry (b). c, Typical optical image captured during a bulging test. The blue line indicates the edge of the tube for determination of the deflection. Scale bar: 0.5 mm.

7. UV-VIS Absorbance spectra of used analytes

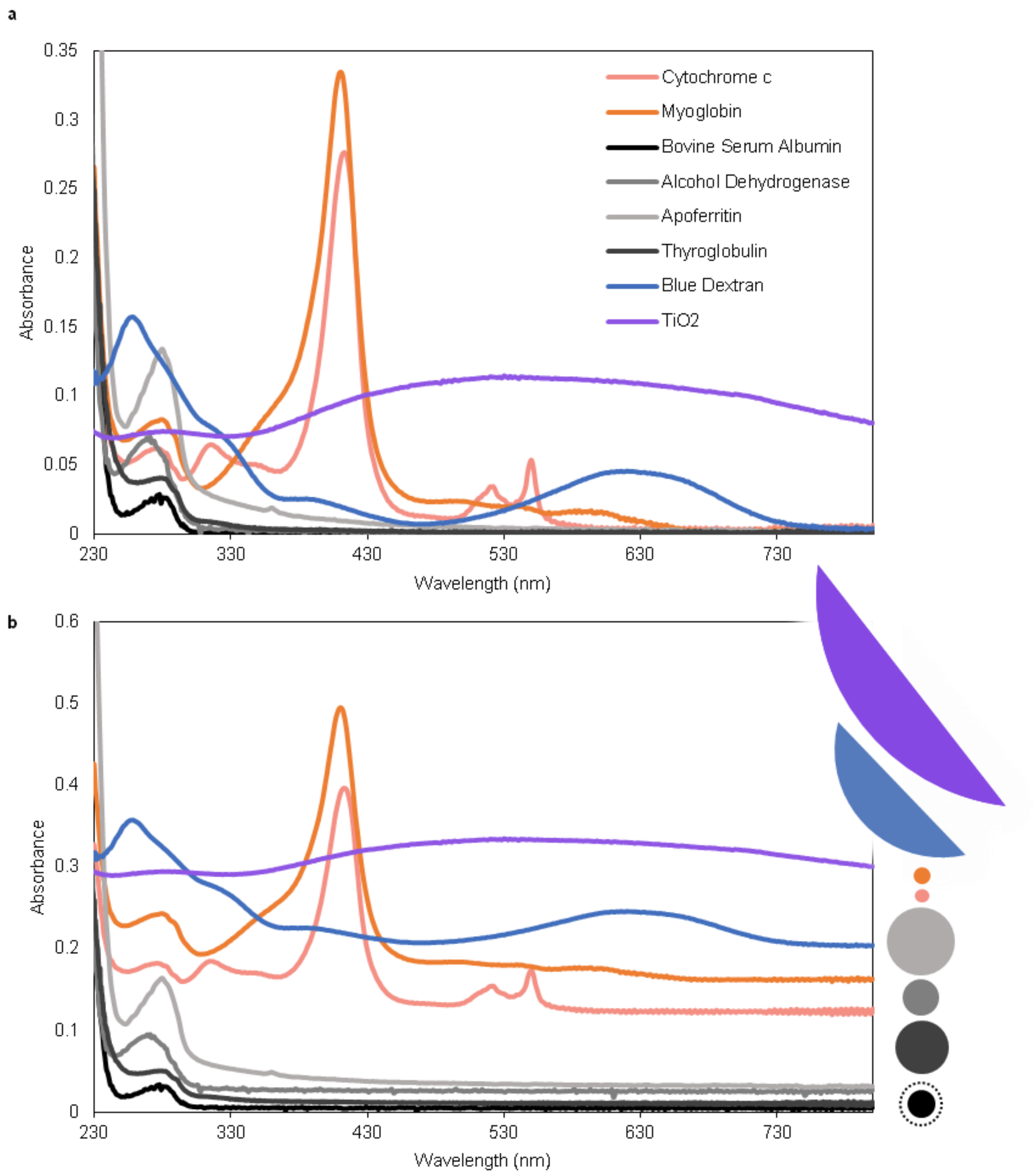

Supplementary Figure 4 UV-VIS absorbance spectra of proteins used in diffusion experiments.

a, Absorbance spectra in the UV-VIS range of model proteins used in this work to investigate separation performance. The displayed spectra were recorded at 0.46 mg mL^{-1}; concentrations that would be present after total equilibrium between retentate and permeate solution has been reached. b, For clarity, absorbance spectra were shifted along the y-axis and the respective hydrodynamic sizes are indicated by correspondingly colored circles (right). A larger size of bovine serum albumin reported in the literature[4] is represented by the dotted circle.

8. Non – perforated nanomembrane diffusion experiment with myoglobin.

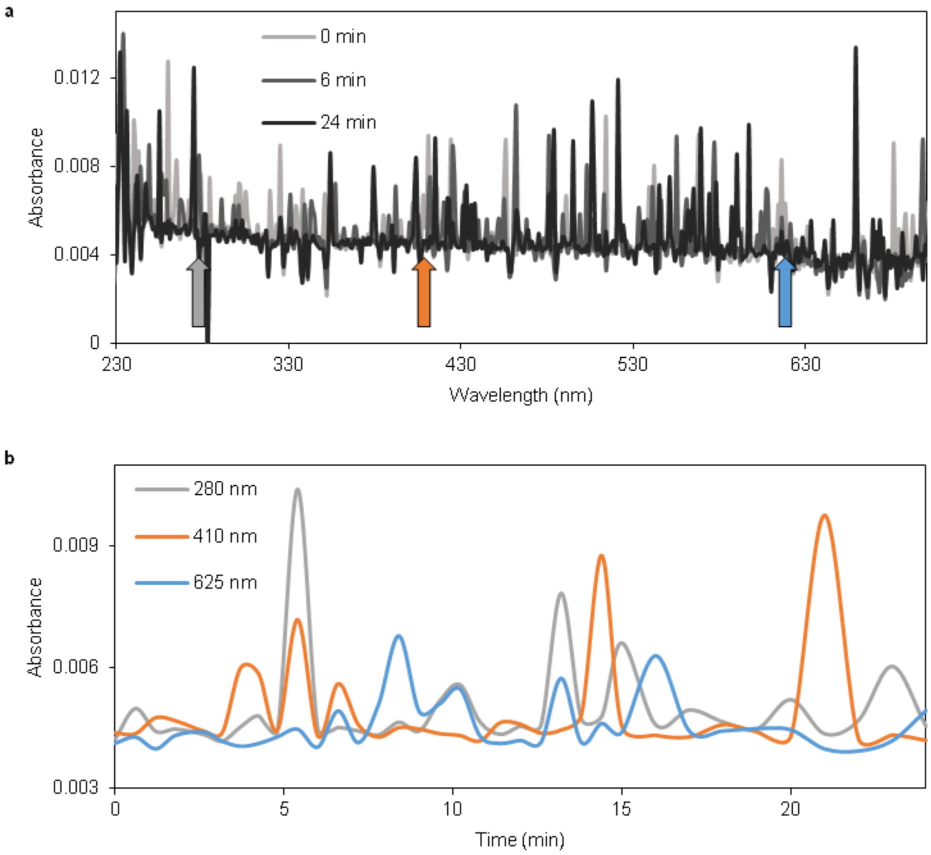

Supplementary Figure 5 Permeability assay with a dense nanomembrane.

a, UV-Vis absorbance spectra of the permeate solution at different points in time. Spectra are changing from gray to black with increasing time. Arrows indicate selected wavelengths plotted versus time in graph b. b, Absorbance of the permeate solution over time at selected wavelengths 280 nm, 410 nm (characteristic absorbance peak maximum of myoglobin) and 625 nm (characteristic absorbance peak maximum of Blue Dextran).

9. Apoferritin diffusion experiment

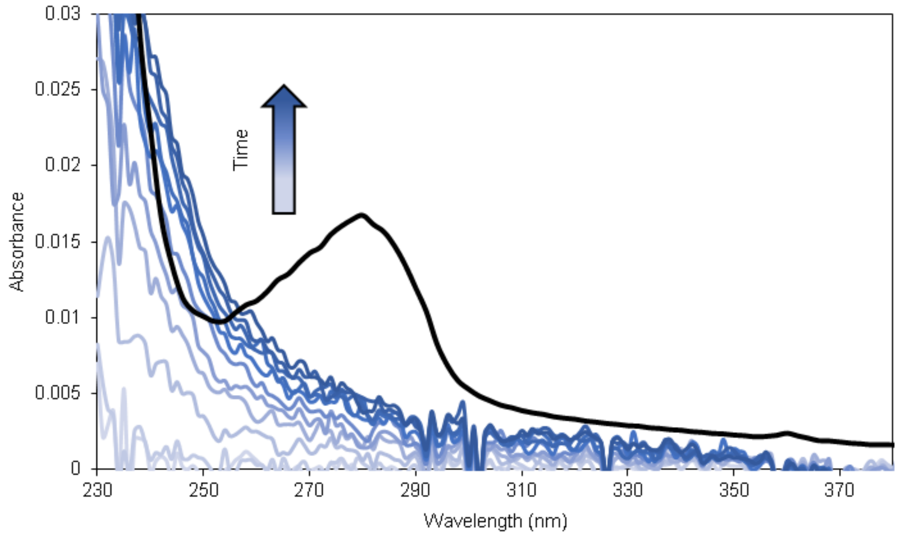

Supplementary Figure 6: Diffusion experiment with apoferritin solution.

UV absorbance spectra of the permeate side over time from light to dark blue with a 1 mg mL^{-1} apoferritin solution. The protein solution was dialyzed with a commercial dialysis membrane before use. The black spectrum represents the absorbance of the protein solution used on the retentate side.

10. Zisman plot

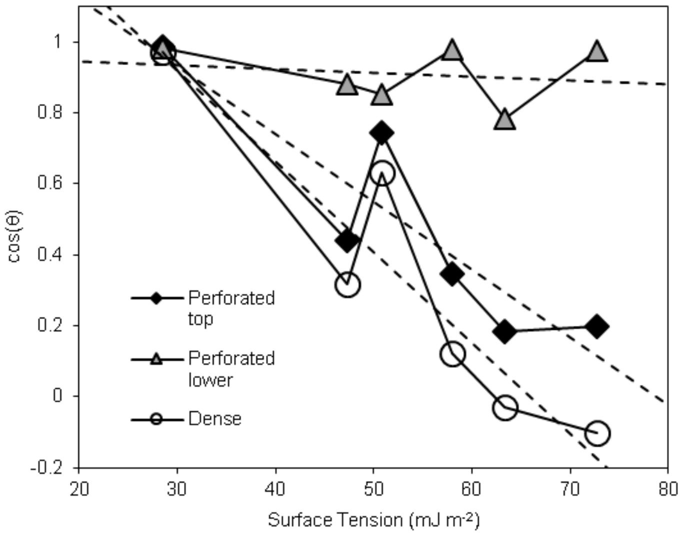

Supplementary Figure 7: Zisman plot of the tested liquids and surfaces.

The contact angle of all tested liquids with the respective surface (cos (θ)) versus the surface tension of the liquid. Extrapolation of the linear regressions to cos (θ) = 1 estimates the surface free energies of the tested surfaces. Results are summarized in Supplementary table 1.

11. Feature detection and pore size distribution from SEM images

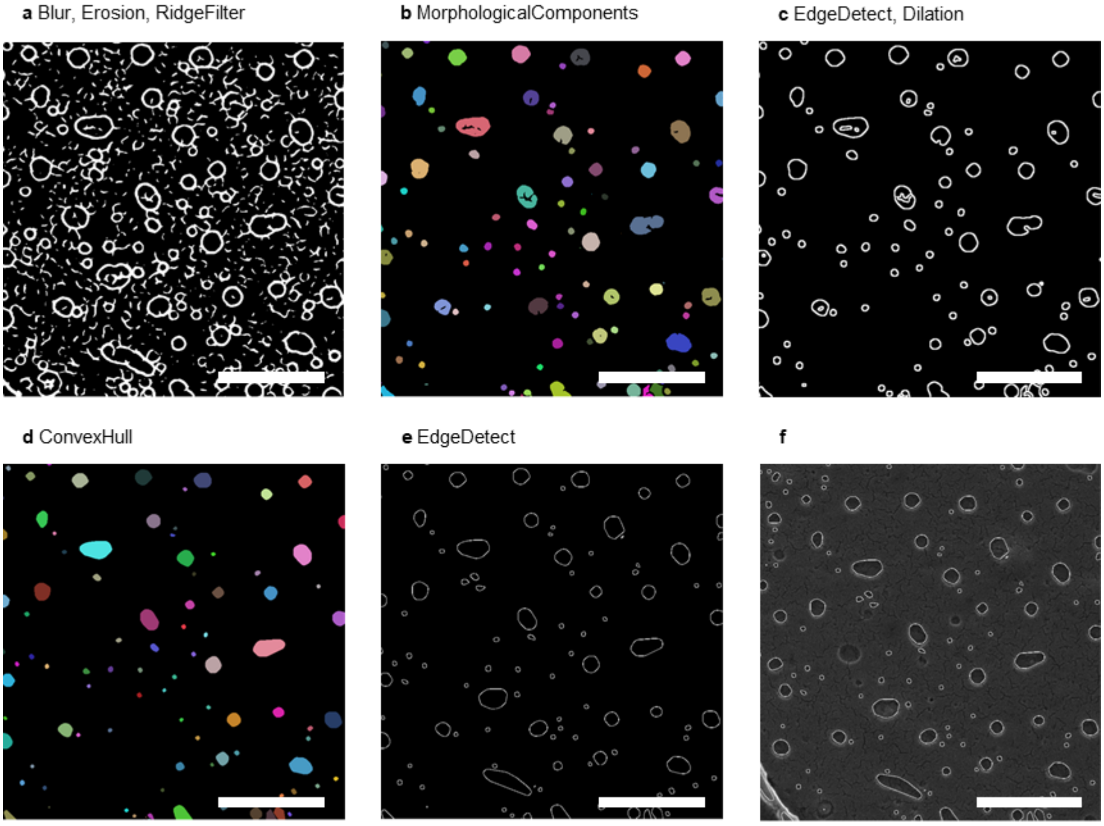

Supplementary Figure 8: Visualized intermediate steps from the processing of an SEM image.

a-d, The filters sequentially applied to the SEM image were visualized for a representative subsection. The detected and amplified edges of the circular features (a) served as boundaries to group the image into the morphological components (b) from which the large ones (e.g. the background) were neglected. The edges of the components were broadened until the encompassed area matched the pore area (c) and these areas were selected as convex features (d) from which the radii of disks with equivalent area were calculated. e, The edges of the detected components (as shown in d) provide better visibility of the result and were superimposed onto the original image in (f). Note that the depicted white lines are part of and contribute to the areas used for calculation. Scale Bars are 1 µm.

12. Pore size distributions obtained from SEM images

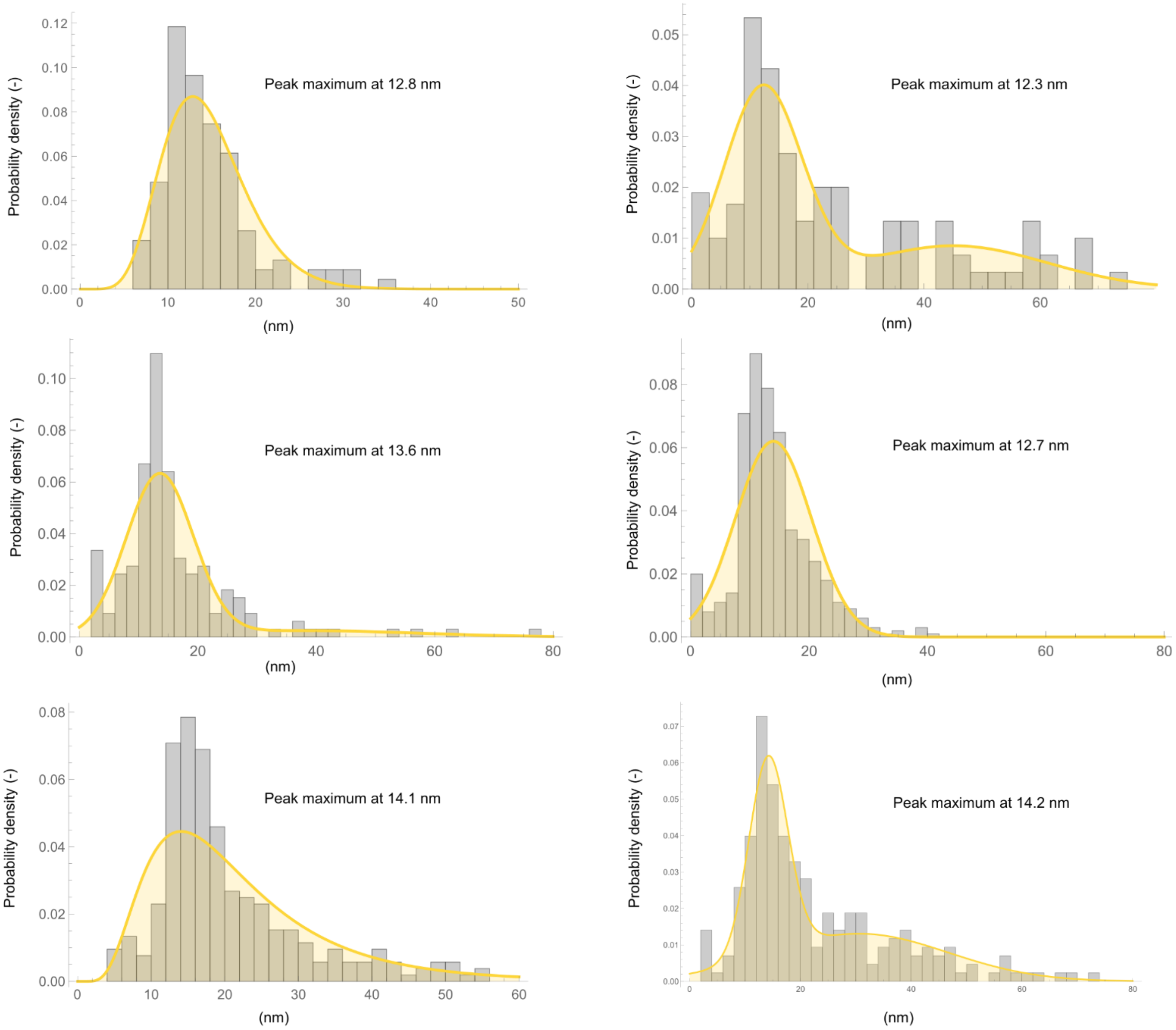

Supplementary Figure 9 Pore size distributions from various membranes.

An average value of the peak maximum of 13.1 ± 0.9 nm was determined from 7 different membranes which exhibited the desired permeability characteristics.

Where adequate, a second normal distribution was fit to the data representing the detected population of circular depressions that are considerably larger in size and would distort the probability density function of the pores.

Supplementary Table 1: Surface free energies of the probed surfaces, wettability of several conventional materials[a] used in protein separations and surface tensions of the test liquids[b].

	Critical / Total	Dispersive	Positive	Negative	$Cos(\theta_{Water})$
	(mJ m^{-2})				(-)
Dense	26.7	29.6	0.0	0.7	-0.10
Perforated top	26.3	32.0	2.3	1.8	0.20
Perforated lower	-35.5	32.4	22.6	12.9	0.97
Cellulose acetate					0.59
Poly(methyl methacrylate)					0.34
Poly(ethylene terephthalate)					0.16
Polystyrene					-0.02
Toluene	28.5	28.5	0	0.7	
Ethylene glycol	48	29	3.0	30.1	
Diiodomethane	50.8	50.8	0	0	
Formamide	58	39	2.3	39.6	
Glycerol	63.4	37	3.9	57.4	
Water	72.8	26.4	25.5	25.5	

[a] Values were taken from[5]

[b] Values were taken from[6]

Supplementary Table 2: Hydrodynamic radii (R_H) and isoelectric points (IEP) of compounds used in diffusion experiments with references.

Compound	R_H (nm)	Ref.	IEP (pH)	Ref.
Cytochrome c	1.78	7	10.04	8
Myoglobin	2.12	7	6.85 / 7.33	9
Bovine serum albumin	5.15 (4.5)	4(10)	4.9	11
Alcohol dehydrogenase	4.55	12	5.4-5.8	13,*
Apoferritin	6.73	14	4	15
Thyroglobulin	8.58	14	4.5-5.0	16
Blue Dextran	26.89	17	7	18

[*] Data from product information provided by Sigma-Aldrich.

13. Supplementary References

1. Small, M. K. & Nix, W. d. Analysis of the accuracy of the bulge test in determining the mechanical properties of thin films. *J. Mater. Res.* **7,** 1553–1563 (1992).
2. ZISMAN, W. A. Relation of the Equilibrium Contact Angle to Liquid and Solid Constitution. in *Contact Angle, Wettability, and Adhesion* **43,** 1–51 (AMERICAN CHEMICAL SOCIETY, 1964).
3. Good, R. J. & Oss, C. J. van. The Modern Theory of Contact Angles and the Hydrogen Bond Components of Surface Energies. in *Modern Approaches to Wettability* 1–27 (Springer, Boston, MA, 1992). doi:10.1007/978-1-4899-1176-6_1
4. Babcock, J. J. & Brancaleon, L. Bovine serum albumin oligomers in the E- and B-forms at low protein concentration and ionic strength. *Int. J. Biol. Macromol.* **53,** 42–53 (2013).
5. Van Oss, C. J., Good, R. J. & Chaudhury, M. K. Additive and nonadditive surface tension components and the interpretation of contact angles. *Langmuir* **4,** 884–891 (1988).
6. Oss, C. J. van. *Interfacial Forces in Aqueous Media*. (CRC Press, 1994).
7. Wilkins, D. K. *et al.* Hydrodynamic Radii of Native and Denatured Proteins Measured by Pulse Field Gradient NMR Techniques. *Biochemistry (Mosc.)* **38,** 16424–16431 (1999).
8. Barlow, G. H. & Margoliash, E. Electrophoretic Behavior of Mammalian-type Cytochromes c. *J. Biol. Chem.* **241,** 1473–1477 (1966).
9. Radola, B. J. Isoelectric focusing in layers of granulated gels. *Biochim. Biophys. Acta BBA - Protein Struct.* **295,** 412–428 (1973).
10. Böhme, U. & Scheler, U. Effective charge of bovine serum albumin determined by electrophoresis NMR. *Chem. Phys. Lett.* **435,** 342–345 (2007).
11. Conway-Jacobs, A. & Lewin, L. M. Isoelectric focusing in acrylamide gels: Use of amphoteric dyes as internal markers for determination of isoelectric points. *Anal. Biochem.* **43,** 394–400 (1971).

12. Potschka, M. Universal calibration of gel permeation chromatography and determination of molecular shape in solution. *Anal. Biochem.* **162,** 47–64 (1987).

13. Kidman, G., Park, H. & Northrop, D. B. Pressure stability of proteins at their isoelectric points. *Protein Pept. Lett.* **11,** 543–546 (2004).

14. Axelsson, I. Characterization of proteins and other macromolecules by agarose gel chromatography. *J. Chromatogr. A* **152,** 21–32 (1978).

15. Petsev, D. N. & Vekilov, P. G. Evidence for Non-DLVO Hydration Interactions in Solutions of the Protein Apoferritin. *Phys. Rev. Lett.* **84,** 1339–1342 (2000).

16. Heidelberger, M. & Pedersen, K. O. THE MOLECULAR WEIGHT AND ISOELECTRIC POINT OF THYROGLOBULIN. *J. Gen. Physiol.* **19,** 95–108 (1935).

17. Armstrong, J. K., Wenby, R. B., Meiselman, H. J. & Fisher, T. C. The Hydrodynamic Radii of Macromolecules and Their Effect on Red Blood Cell Aggregation. *Biophys. J.* **87,** 4259–4270 (2004).

18. Friscia, S., Turchi, S. L. & Hepfer, C. E. The influence of pH on electrophoretic mobility, a laboratory investigation. *Biochem. Educ.* **20,** 41–42 (1992).
